# Combined Inhibition of Epidermal Growth Factor Receptor and Cyclooxygenase-2 Leads to Greater Anti-tumor Activity of Docetaxel in Advanced Prostate Cancer 

**DOI:** 10.1371/journal.pone.0076169

**Published:** 2013-10-14

**Authors:** Jianzhong Lin, Hongfei Wu, Hui Shi, Wei Pan, Hongbo Yu, Jiageng Zhu

**Affiliations:** 1 Department of Urology, BenQ Medical Center, Nanjing Medical University, Nanjing, China; 2 Laboratory of Reproductive Health, Jiangsu Institute of Planned Parenthood Research, Nanjing, China; 3 Department of Oncology, the Second Hospital of Nanjing Jiangning, Nanjing, China; 4 Department of Urology, Nanjing First Hospital, Nanjing Medical University, Nanjing, China; Roswell Park Cancer Institute, United States of America

## Abstract

The epidermal growth factor receptor (EGFR) and cyclooxygenase-2(COX-2) play a critical role in disease progression, relapse and therapeutic resistance of advanced prostate cancer (PCa). In this paper, we evaluated, for the first time, the therapeutic benefit of blocking EGRF and/or COX-2 (using gefitinib and NS-398, respectively) in terms of improving the efficacy of the conventional clinical chemotherapeutic drug docetaxel in vitro and vivo. We showed that EGFR and COX-2 expression was higher in metastatic than non-metastatic PCa tissues and cells. Docetaxel, alone or in combination with gefitinib or NS-398, resulted in a small decrease in cell viability. The three drug combination decreased cell viability to a greater extent than docetaxel alone or in combination with gefitinib or NS-398. Docetaxel resulted in a modest increase in apoptotic cell in metastatic and non-metastatic cell lines. NS-398 markedly enhanced docetaxel-induced cell apoptosis. The combination of the three drugs caused even more marked apoptosis and resulted in greater suppression of invasive potential than docetaxel alone or in association with gefitinib or NS-398. The combination of all three drugs also resulted in a more marked decrease in NF-ΚB, MMP-9 and VEGF levels in PC-3M cells. These in vitro findings were supported by in vivo studies showing that docetaxel in combination with gefitinib and NS-398 was significantly more effective than any individual agent. Based on previous preclinical research, we conclude that simultaneously blocking EGFR and COX-2 by gefitinib and NS-398 sensitizes advanced PCa cells to docetaxel-induced cytotoxicity.

## Introduction

Prostate cancer (PCa) is the most common malignancy, and is one of the leading causes of death among elderly men [1]. The response rate to radical prostatectomy and hormone ablation therapy is high in patients diagnosed with localized and androgen-dependent cancers. However, the progression to hormone-refractory prostate cancers (HRPC) and/or bone metastases is associated with disease relapse and poor patient survival [2-4]. Indeed, progression of prostate cancer to androgen independence remains a primary barrier to improving patient survival as it is associated with complex underlying cellular changes. 

Docetaxel is considered as the standard chemotherapeutic agent for patients with HRPC and in those with clinical evidence of metastases. It has been reported to improve quality of life and offers pain relief, but it is associated with minimal a median survival rate of only 12 to 19 months from the start of treatment. This highlights the need for trials investigating how to optimize conventional chemotherapeutic regimens in patients with HRPC or advanced PCa. 

Investigating the molecular mechanisms that underlie PCa progression, will help to identify the putative therapeutic target genes involved in apoptosis. It will also help to elucidate the mechanism responsible for growth and cell signaling [5-8]. EGFR and COX-2 have both been shown to contribute to sustained growth in advanced HRPC in either the absence or presence of low concentrations of androgen [9-11]. 

EGFR is frequently overexpressed in human cancers. Preclinical data suggest that the EGFR signaling pathways activate androgen receptors under conditions of clinical androgen deprivation. This is linked to the transition from the androgen-responsive to the hormone-refractory phenotype, resulting in a more aggressive clinical outcome [10,11]. EGFR is therefore, assumed to be of primary therapeutic importance, due to its overexpression in advanced PCa and its role as a drug target. Previous studies have demonstrated that activation of EGFR enhances the ability of androgen receptors to increase PCa proliferation. BY contrast, inhibition of EGFR was shown to improve the efficiency of docetaxel in the treatment of metastatic PCa [12,13]. 

Cyclooxygenases catalyze the formation of prostaglandins involved in tumor initiation and/or progression. COX-2 has been shown to promote inflammation, which may directly contribute to the development of PCa [14]. It has also been shown that COX-2-induced PGE2 activates cell signaling involved in proliferation and thereby directly promotes tumor cell growth. Other studies have demonstrated that COX-2 is overexpressed in PCa and that its level of expression correlates with Gleason score, cancer progression and recurrence [15,16]. In recent years, COX-2 inhibitors in combination with chemotherapeutic drugs have been evaluated in the treatment of advanced PCa. These agents significantly increase the efficacy of androgen withdrawal and promote the resolution of skeletal lesions [17,18]. It is generally accepted that COX-2 contributes to PCa and there is mounting evidence to suggest that COX-2 inhibitors may be beneficial in the treatment of PCa. 

Previous studies have confirmed that high COX-2 expression in PCa is correlated with docetaxel resistance, and that inhibition of COX-2 significantly slows tumor growth and improves the efficacy of docetaxel [19,20]. Based on these observations, it is possible that the addition of selective inhibitors of EGFR and COX-2 may represent a novel therapeutic approach for improving the treatment of metastatic PCa. In the present study, we hypothesized that simultaneous blockade of EGFR and COX-2 pathways using gefitinib and NS-398 might improve the cytotoxic effects of docetaxel in advanced PCa in vitro and vivo. The anti-proliferative, anti-invasive, and apoptosis induction effects of the three agents, alone and in combination, were determined in vitro and vivo and the underlying molecular mechanisms were investigated.

## Materials and Methods

### Ethics statement

All experiments involving animals were conducted with the approval of Nanjing Medical University Ethics Committee. The clinical investigation was approved by the Ethics Committee of Nanjing Medical University. Written informed consent was obtained for all subjects prior to participation in the study.

### Tumor samples and cell lines

PCa samples from 37 primary adenocarcinoma cases were included the study. In all cases two independent pathologists reviewed the specimens to exclude other pathological types. All samples were obtained by transurethral resection of the prostate, radical prostatectomy or needle-biopsy at the Nanjing BenQ Hospital and Nanjing First Hospital, between 2010 and 2012. Surgical staging of tumors was performed according to Jewett–Whitmore criteria. The median age of the patients was 69 years (range 58 to 79 years).

The human prostate cancer cell lines LNCaP, PC-3M and DU-145 used in the study were purchased from the American Type Culture Collection and were maintained in RPMI-1640 culture medium containing 10% fetal bovine serum, 26 mmol/L NaH_2_CO_3_ (pH 7.4), 1% L-glutamine, and antibiotics (100 IU/mL penicillin-100μg/mL streptomycin) at 37°C in 5% CO_2_. RPMI 1640 and other culture materials were supplied by Gibco. 

### Reagents and antibodies

Gefitinib, NS-398, docetaxel and MTT were purchased from Sigma. Annexin V-FITC was supplied by Gibco. Antibodies against human VEGF and MMP-9 were obtained from R&D System and Bioworld, respectively. Antihuman monoclonal COX-2, monoclonal EGFR and monoclonal VEGF antibodies were obtained from Santa Cruz. Antibody against human NF-ΚB P65 was purchased from Abcam. Monoclonal antibodies to GAPDH (Bioworld) and β-actin (Sigma) were used as experimental controls. 

### Immunohistochemistry

Histological sections (4 μm) fixed in 10% formalin and embedded in paraffin were used for immunohistochemical staining. Prior to a primary antibody staining, the slides were pretreated with citric acid or ethylenediamine tetraacetic acid buffer in a pressure cooker for antigen retrieval. All primary antibodies used in the study were biotinylated monoclonal antibodies. 

Endogenous peroxidase activity was quenched using 3% H_2_O_2_ blocking reagent for 10 min. The slides were then incubated with a primary antibody at 4°C overnight, prior to immunostaining with avidin-biotin peroxidase complex (100: l). The slides were stained with diaminobenzidine according to the manufacturer’s protocol (DAKO, CA). The slides were rinsed three times with phosphate buffered saline after each staining step. The sections were stained with a 1:200 dilution of the antibody against EGFR (Santa Cruz Biotechnology, Santa Cruz, CA, USA), and 1:100 dilution of the antibody against COX-2 (Santa Cruz Biotechnology, Santa Cruz, CA,USA) respectively. 

The stained slides were evaluated quantitatively or semi-quantitatively by two independent pathologists who were blind to the clinical data. The median number of tumor cells staining positive for COX-2 and EGFR was 10% in each case. The percentage of positive cells stained with special antibody observed by two pathologists were consistent and mean values were determined.

### Culture and cell viability Assays

The effects of individual agents, and of double and triple combinations of agents on PC-3M and DU-145 cell lines were determined after 48 h exposure to 3-(4,5-dimetylthiazol-2-yl) -2,5-diphenyltetrazolium bromide (MTT). Reproducibility was confirmed in three independent experiments. To test the viability after exposure to gefitinib, NS-398 and docetaxel alone or in combination, cells were seeded on 96-well plates at a density of approximately 5×10^4^ /mL (in 100 μL of medium per well) and were incubated overnight at 37°C. Based on previous studies, we chose concentrations of 20 μmol/L gefitinib,100 μmol/L) NS-398 and 0.01μmol/L docetaxel for all assays. Cells exposed to DMSO were used as untreated controls. 

### In vitro invasion assay

The invasive potential of prostate cancer cells was estimated by their ability to penetrate a Matrigel invasion chamber comprising an 8 μm pore size polyethylene terephthalate membrane, and a thin layer of Matrigel matrix. PC-3M and DU-145 control cells or cells previously exposed for 24 h to gefitinib (10 μmol/L), NS-398 (50 μmol/L), or docetaxel (0.005 μmol/L) alone or in combination were used in the cell invasion assay. In each experiment, 5×10^4^/mL cells per well, in medium without drugs (control) or containing the drugs, were loaded into the top of the Matrigel cell invasion chamber, or into the control insert chamber without Matrigel, according to the manufacturer’s instructions. After incubation for 24 h at 37°C, the invading cells reaching the lower chamber were stained with a crystal violet,, and counted by phase-contrast microscopy. The number of invading cells was estimated as the mean number invading through the Matrigel insert membrane in five random microscope fields.

### Flow cytofluorometric analyses

The cells were exposed to gefitinib (20 μmol/L), NS-398(100 μmol/L), or docetaxel (0.01 μmol/L), alone or in combination. Following 24 h incubation, both the growth and wash medium were saved, and cells were harvested with trypsin. Supernatants were removed and pellets were resuspended in 400 μL of propidium iodide (PI) solution (50 μg/mL PI, 0.1% Triton X-100, and 0.1% sodium citrate in PBS). Samples were then incubated overnight at 4°C in the dark prior to analysis. Flow cytometry was quantified to determine the percentage of cells containing apoptotic, necrotic cells.

### Reverse transcription and real-time PCR

PC-3M and DU-145 cells grown in 1% FBS were exposed for 24 h to gefitinib (20 μmol/L), NS-398 (100 μmol/L), or docetaxel (0.01 μmol/L) alone or in combination. Cells exposed to DMSO as vehicle, were used as controls. Total RNA was isolated using Trizol reagent. One microgram of RNA was reverse transcribed using a reverse transcription system (Invitrogen, Carlsbad, CA) according to the manufacturer’s instructions. Real-time PCR was used to quantify mRNA expression. The primer sequences used are as follows: MMP9 (forward: 5’-TC GAACTTTGACAGCGACAAGA-3’ and reverse 5’-TCAGGGCGAGGACCATAGAG’-3), NF-ΚB (forward:5’-TGGACCGCTTGGGTAACTCT-3’and reverse 5’-GGCTATTGCTCATCA TGGCTAG-3’), VEGF (forward:5’-CTATCAGCGCAGCTACTGCCAT-3’and reverse 5’- GCACACAGGATGGCTTGAAGAT-3’). GADPH was used as an internal control to correct potential variation in RNA loading. Target and GADPH genes were run under the same conditions. All reactions were undertaken in a 20 μL volume containing the sample cDNA, TaqMan fast universal PCR mastermix, primers and probes. The PCR conditions were 40 to 45 cycles at 95°C for 15 s followed by 1 min cycles at 59 to 65 °C.

### Western blot assay

PC-3M and DU-145 cells exposed to the drugs described above were harvested by scraping from the wells and washings twice with cold PBS. The cell pellet was suspended in 125 mmol/L Tris-HCl (pH 6.8), sonicated for 10 s prior to adding an equal volume of 4% SDS. The lysates were boiled for 10 min. Protein concentration was determined using a bicinchoninic acid protein assay (Pierce, Rockford, IL). 

The proteins were separated by SDS-PAGE, transferred to nitrocellulose, blocked, and incubated with primary antibodies as follows: VEGF diluted 1:1000 (Santa Cruz Biotechnology, Inc. Santa Cruz, CA, USA), MMP-9 (4 μg/mL, R&D Systems), NF-ΚB ((Abcam Ltd., UK).), and β-actin 1:10000 (Sigma) /GADPH 1:10000 (Bioworld). The membrane was then washed and incubated with secondary antibodies (Bioworld) conjugated with peroxidase. Protein detection was undertaken using a chemiluminescence detection system (Thermo).

### Animal experiments

Four to 6-week-old BALB/c nu/nu mice were obtained from the SLAC Laboratory Animal Co. Ltd (Shanghai, China). Tumors were grown bilaterally in athymic nude mice by subcutaneous injection of 5×10^4^-10^5^ PC-3M cells (suspended in PBS) per animal. Well-established tumors 0.2 cm^3^ in diameter were detected after 7 days. The mice were then randomized into the following groups of six: untreated controls, gefitinib (50 mg/kg, qd, p.o.), NS-398 (200 mg/kg, qd, p.o), docetaxel (0.05 mg/kg, qd, p.o), gefitinib and docetaxel, NS-398 and docetaxel, gefitinib , NS-398 and docetaxel. 

Tumor measurements were performed throughout the course of treatment and observation periods. Tumor volume was measured using the formula: π/6×larger diameter*×*(smaller diameter)^2^. Surgery was performed under ketamine/xylazine anesthesia, and every effort was made to minimize suffering. 

### Statistical Analysis

Statistical analyses was undertaken using SPSS version 16.0 software. Data are presented means and standard deviations (±SD). The index for combination treatment in PC-3M and DU-145 cells was calculated using CalcuSyn software. According to this method, CI values of <1, 1 and >1 respectively represented synergy, additivity and antagonism [21].

In vitro and in vivo data were analyzed using Student's t-test. EGFR and COX-2 expression in non-metastatic and metastatic PCa tissues was compared using the Pearson’s chi-square test.. Values of p <0.05 were considered statistically significant.

## Results

### EGFR and COX-2 protein levels in prostate cancer tissues

**Figure 1:**
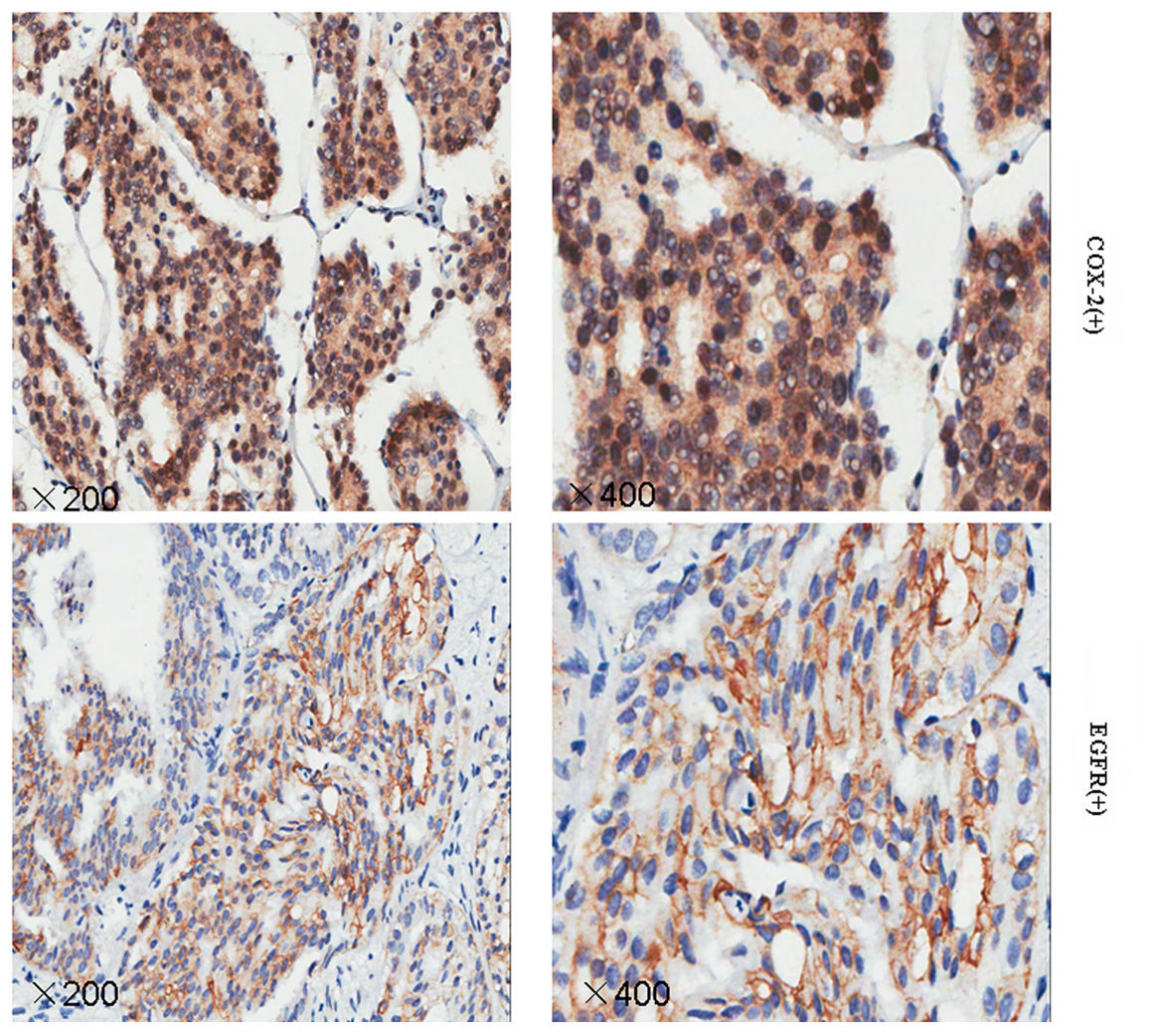
Immunohistochemical analyses of EGFR and COX-2 expression levels in prostate cancer tissues. Sections were examined under a microscope, and immunoreactivity was indicated by dark brown staining. Representative sections of prostate cancer tissue samples obtained at original magnifications of ×200 and ×400.

EGFR expression was positive in seven non-metastatic PCa tissues (43.8%) and in 18 (85.7%) tissue samples from patient with metastatic PCa (*P*<0.01). Intensity staining for COX-2 was seen in 18 metastatic (85.7%) and nine non-metastatic PCa tissues (56.3%; P=0.11). EGFR and COX-2 positive coexpression was found in 16 metastatic tissues (76.2%) and in four (25.0%) non-metastatic PCa tissues (P <0.01). The results are shown in Figure 1 and Table 1.

**Table 1:** The positive expression of EGFR and COX-2 in non-metastatic and metastatic prostate cancer.

**Stage**	**n**	**Number (%) patients**			
		**EGFR+COX-2+**	**EGFR+COX-2-**	**EGFR-COX-2+**	**EGFR-COX-2-**
A+B	16	4 (25.0%)	3 (18.8%)	5 (31.2%)	4 (25.0%)
C+D	21	16 (76.2%)	2 (9.5%)	2 (9.5%)	1 (4.8%)

Pathological staging of PCa was defined according to the Whitmore-Jewett staging system as follows

A (incidentally discovered PCa), B (limited PCa), C (invasion to adjacent organs)

D (distant metastases or lymph node involvement).

Jewett stage A and B was defined as non-metastatic and stage C or D as metastatic.

### Baseline expression and activation of EGFR and COX-2 protein in PC-3M and DU-145 cells

**Figure 2:**
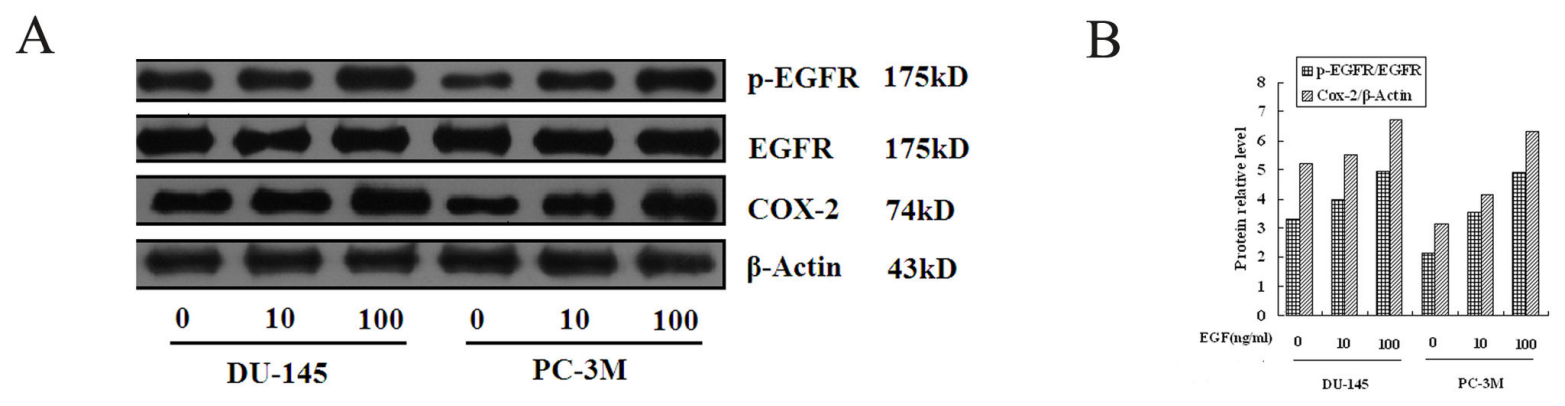
Baseline expression and activation levels of EGFR and COX-2 protein levels in hormone-refractory PCa cells (PC-3M and DU-145). Cells grown in 1% FBS were exposed to 0, 10 and 100 ng/mL EGF for 24 h as described in Materials and Methods. The resulting cellular proteins were subjected to SDS-PAGE and Western blot analysis to determine the protein levels of p-EGFR, EGFR and COX-2 (Figure 2A). Western blot for β-actin is shown as the loading control. The relative protein level for p-EGFR/EGFR and COX-2 is shown in Figure 2B.

The basal level of the total EGFR, p-EGFR and COX-2 was measured in PC-3M and DU-145 cells. We then investigated whether EGF was associated with an increase these levels. The results indicated that activated/phosphorylated EGFR (p-EGFR), EGFR and COX-2 protein were expressed in both cell lines(Figure 2A). p-EGFR and COX-2 expression were significantly increased in the two cell lines by the addition of EGF, but EGFR expression was unchanged. The change in p-EGFR/EGFR ratio was dose dependent (Figure 2B). 

### Anti-proliferative effect induced by docetaxel alone or in combination with gefitinib and NS-398

**Figure 3:**
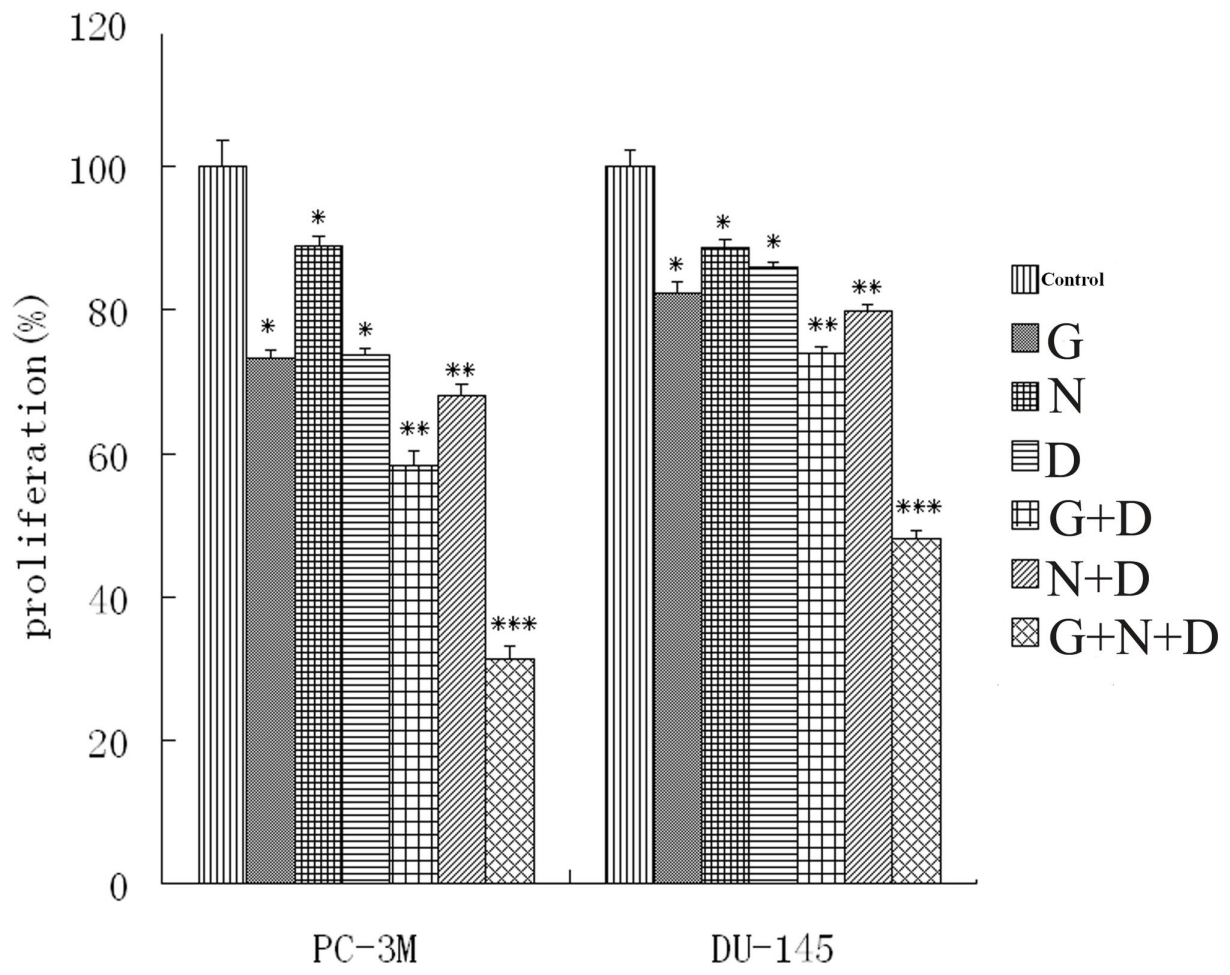
Anti-proliferative effect of docetaxel (D), gefitinib (G), and NS-398 (N) in PC-3M and DU-145 cell lines evaluated by the MTT assay. The concentration-response response for each drug was used to determine the concentration that induced an appropriate anti-proliferative effect for use in combination therapy (data not shown). Cells were exposed to docetaxel (0.01 μmol/L), NS-398 (100 μmol/L) and gefitinib (20 μmol/L) alone or in combination for 24 h. Values are means±SD of four independent experiments. *P <0.05, ** P <0.005 ***, P <0.001 as compared with control values.

MTT assays were used to evaluate the anti-proliferative effect of the three drugs alone or in combinations, in PC-3M and DU-145 cells. In preliminary experiments, we established concentration-response curves for each drug to determine the concentration that produced less than 30% growth inhibition in the PCa cells lines (data not shown). Based on these curves, we determined the that concentrations of docetaxel (0.01 μmol/L), gefitinib (20 μmol/L), or NS-398 (100 μmol/L) were appropriate for use in the combination experiments. Co-incubation with gefitinib or NS-398 slightly enhanced the cytotoxic effect of docetaxel. However, the three drug combination induced a greater than additive effect on cell growth-inhibition in both cell lines which was much more marked than that seen with any one or two drugs combinations (Figure 3). Analysis of combination treatment in PC-3M and DU-145 cells indicated that the combination index for the three agents combination was <1 suggesting a synergistic effect (Table 2 and 3).

**Table 2:** Effect of gefitinib (G) or NS-398 (N) in combination with docetaxel (D) in PC-3M and DU-145 cells.

**Cell lines**	**G (μM)**	**D (nM)**	**Fa**	**CI**	**N (μM)**	**D (nM)**	**Fa**	**CI**
**PC-3M**	10	5	0.215	1.153	50	5	0.211	0.982
	20	10	0.280	1.049	100	10	0.308	0.726
	40	20	0.363	1.118	200	20	0.388	1.023
**DU-145**	10	5	0.186	1.839	50	5	0.264	0.966
	20	10	0.265	1.114	100	10	0.383	0.924
	40	20	0.325	1.092	200	20	0.585	0.956

**Table 3:** Effect of gefitinib (G), NS-398 (N) and docetaxel (D) combination in PC-3M and DU-145 cells.

**Cell lines**	**G (μM)**	**N (μM)**	**Dl (nM)**	**Fa**	**CI**
**PC-3M**	10	50	5	0.302	0.745
	20	100	10	0.465	0.712
	40	200	20	0.661	0.699
**DU-145**	10	50	5	0.264	0.966
	20	100	10	0.423	0.783
	40	200	20	0.621	0.872

Combination treatments with gefitinib (10, 20, and 40 µM), NS-398 (50, 100, and 200 µM), and docetaxel (5, 10, and 20 nM) in PC-3M and DU-145. Cell lines were evaluated by MTT assay.

Fa: Fraction of growth affect of drug-exposed cells compared with controls. CI , combination index. Fa and CI were calculated using CalcuSyn software.

### Cytotoxic effect induced by docetaxel, gefitinib, and NS-398

**Figure 4:**
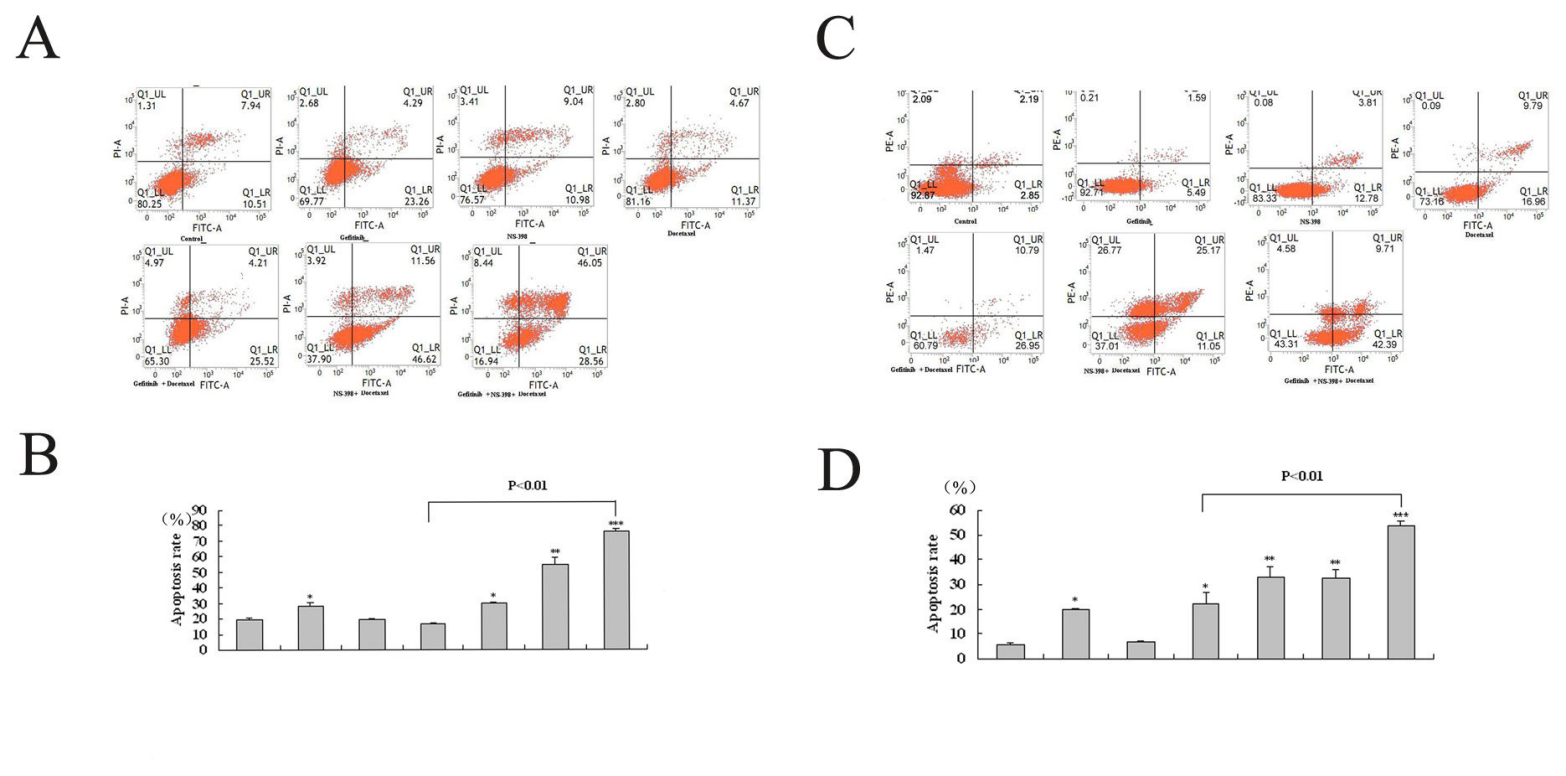
FACS analyses of apoptosis induction effect induced by docetaxel (D, 0.01 μmol/L), NS-398 (N, 100μmol/L) and gefitinib (G, 20μmol/L) alone or in combination following 24 h incubation with PC-3M (A and C) and DU-145 cells (B and D). After incubation with various drugs, the cells were prepared as described in Materials and Methods and apoptotic rate was assessed by FACS analyses. A and C: Representative dot plots illustrate the data near the mean of groups in B and D. B and D. Apoptosis rate was calculated from UR (non-viable apoptotic or necrosis cell rate) and LR (early apoptotic cell rate) association. Representative results from three separate experiments. *P <0.05, ** P <0.005 ***, P <0.001 compared with control values.

Flow cytometric analyses were used to determine cell apoptosis induced by any drug alone or in combination. The number of apoptotic cells was quantified (Figure 4A and C). Both docetaxel and gefitinib, but not NS-398, slightly increased the number of apoptotic cells compared to that seen in untreated PCa cells (Figure 4B and D). Docetaxel in combination with gefitinib or NS-398, was associated with an increase in the number of apoptotic cells compared to docetaxel alone (Figure 4B and D). The three drug combination resulted in a significantly higher numbers of apoptotic cells than any single drug or two drugs combination (Figure 4B and D).

### Loss of invasive ability induced by docetaxel, gefitinib, and NS-398

**Figure 5:**
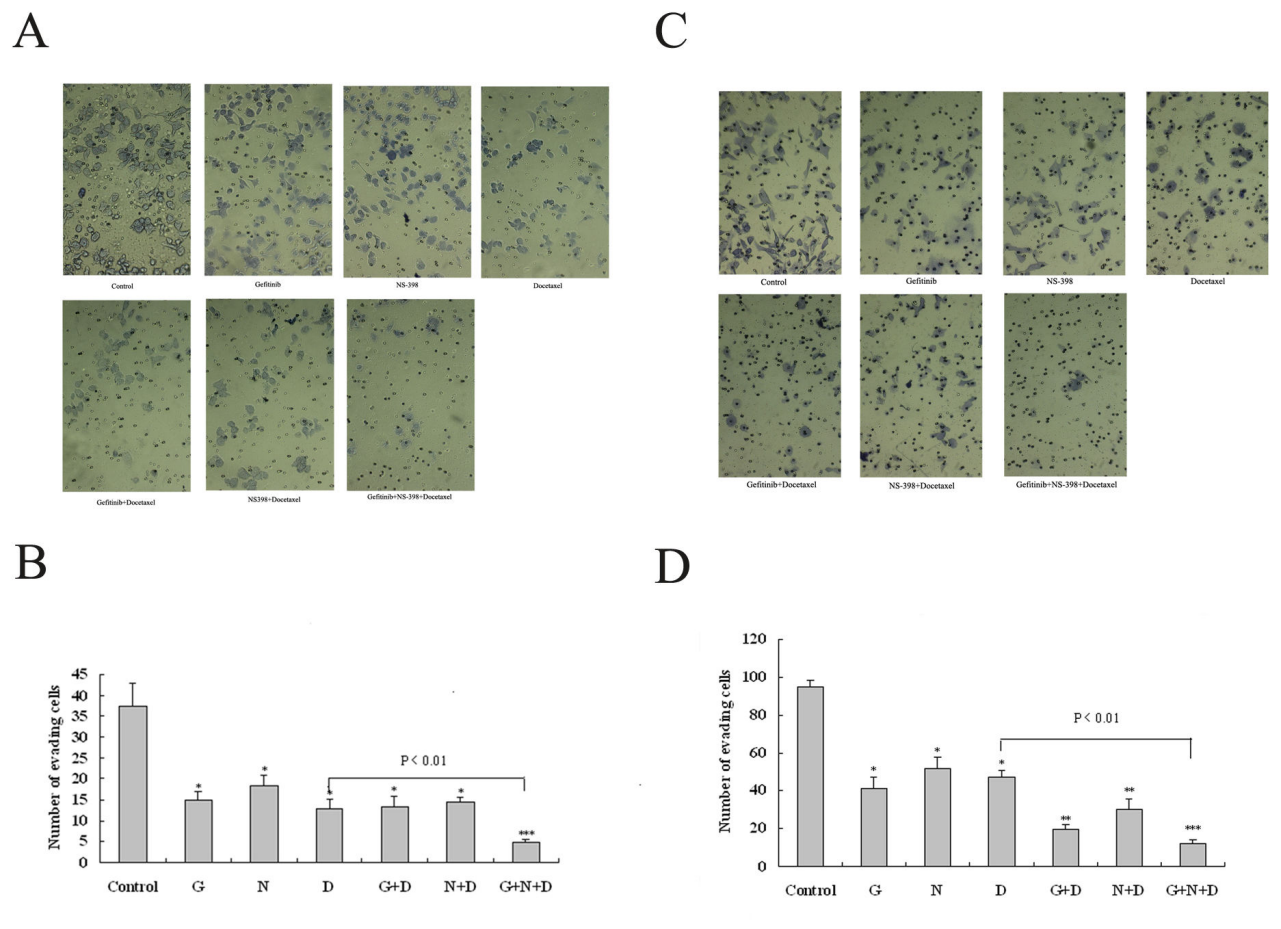
Inhibitory effect induced by docetaxel, gefitinib, and NS-398 on the invasive potential of PC3M and DU-145 cells. The cells were untreated (control) or exposed for 24 h to 0.005 μmol/L docetaxel (D), 10 μmol/L gefitinib (G) or 50 μmol/LNS-398 (N), alone or in combination during in vitro invasion assays, performed using transwell bicameral chambers as described in Materials and Methods. At the end of the 24 h assay, the invading cells were stained and counted by phase-contrast microscopy (x 200). Representative results show the mean number of cells invading through the Matrigel insert membrane in five random microscope fields. *P <0.05, ** P <0.005, ***, P <0.001 compared with control values.

An in vitro invasion assay was used to evaluate the invasive ability in PC-3M and DU145 cells. The invasive potential was determined by calculating the number of cells which penetrated a Matrigel invasion chamber. As shown in Figure 5A and C, exposing cells to 0.005 μmol/L docetaxel, 10 μmol/L gefitinib, or 50 μmol/L NS-398 significantly inhibited their invasive ability (Figure 5B and D).

Exposure to docetaxel in combination with gefitinib or NS-398 markedly reduced the number of DU-145 cells penetrating the Matrigel in comparison with docetaxel alone (Figure 5D), but no difference was seen in the PC-3M cells (Figure 5B). The three drug combination showed a supra-additive inhibitory effect on cell invasion ability which was stronger than any two-drug combination (Figure 5B and D).

### NF-ΚB, MMP-9 and VEGF expression changes induced by docetaxel, gefitinib, and NS-398

**Figure 6:**
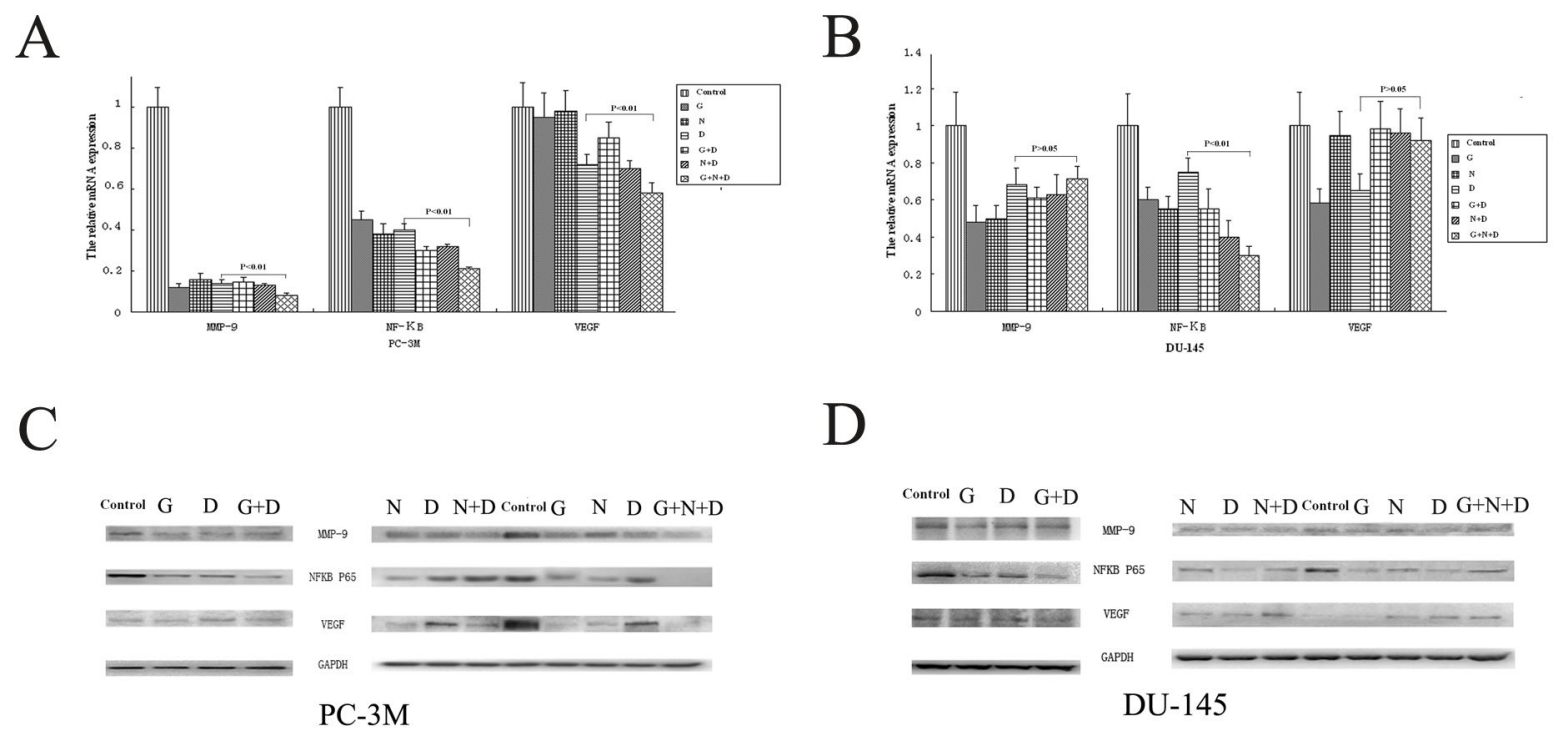
The effects of Docetaxel, gefitinib, and NS-398 on NF-KB, MMP-9 and VEGF mRNA and protein levels in PC-3M and DU-145 cells. Cells grown in 1% FBS were exposed to 0.01 μmol/L docetaxel (D), 20 μmol/L gefitinib (G) or100 μmol/LNS-398 (N), alone or in combination, or with DMSO as control for 24 h. NF-KB, MMP-9 and VEGF mRNA (A and B) and protein levels (C and D) were measured as described in Materials and Methods. Values are reported as the mean±SD of three independent experiments.

Protein and mRNA and were quantified to investigate whether NF-ΚB, MMP-9 and VEGF, were involved in the cellular responses to docetaxel, gefitinib or NS-398. The results in Figure 6A and 6B, show that docetaxel, gefitinib and NS-398 each separately downregulated NF-ΚB, MMP-9 and VEGF mRNA expression in both PCa cell lines. NF-ΚB mRNA expression was more markedly down-regulated when cells were exposed to drug combinations. The three drug combinations showed a stronger inhibitory effect than the two drug combinations (Figure 6A and B). 

Neither gefitinib nor NS-398 had any additive effect on docetaxel-induced downregulation of MMP-9 and VEGF mRNA expression (Figure 6A and B). However, co-incubation with all three agents resulted in more marked inhibition of MMP-9 and VEGF levels compared to single or two drugs combination in PC-3M cells (Figure 6A) but not in DU-145 cells (Figure 6B) ,

Western blotting was undertaken to estimate the protein levels of NF-ΚB MMP-9 and VEGF after exposure to each of the drugs. As shown in Figure 6C and 6D, the addition of docetaxel to gefitinib and/or NS-398 in double and triple combination changed NF-ΚB, MMP-9 and VEGF protein levels in both cell lines. These results are generally consistent with the mRNA changes except for changes in NF-ΚB protein after exposure to a combination of docetaxel and NS-398,(Figure 6C and D). Taken together, these results suggest that the beneficial response of PC-3M cells to exposure to the docetaxel, gefitinib and NS-398 combination, is influenced by a reduction in NF-ΚB, MMP-9 and VEGF (Figure 6C).

### Effects of gefitinib, NS-398 and docetaxel on prostate tumor growth in vivo

**Figure 7:**
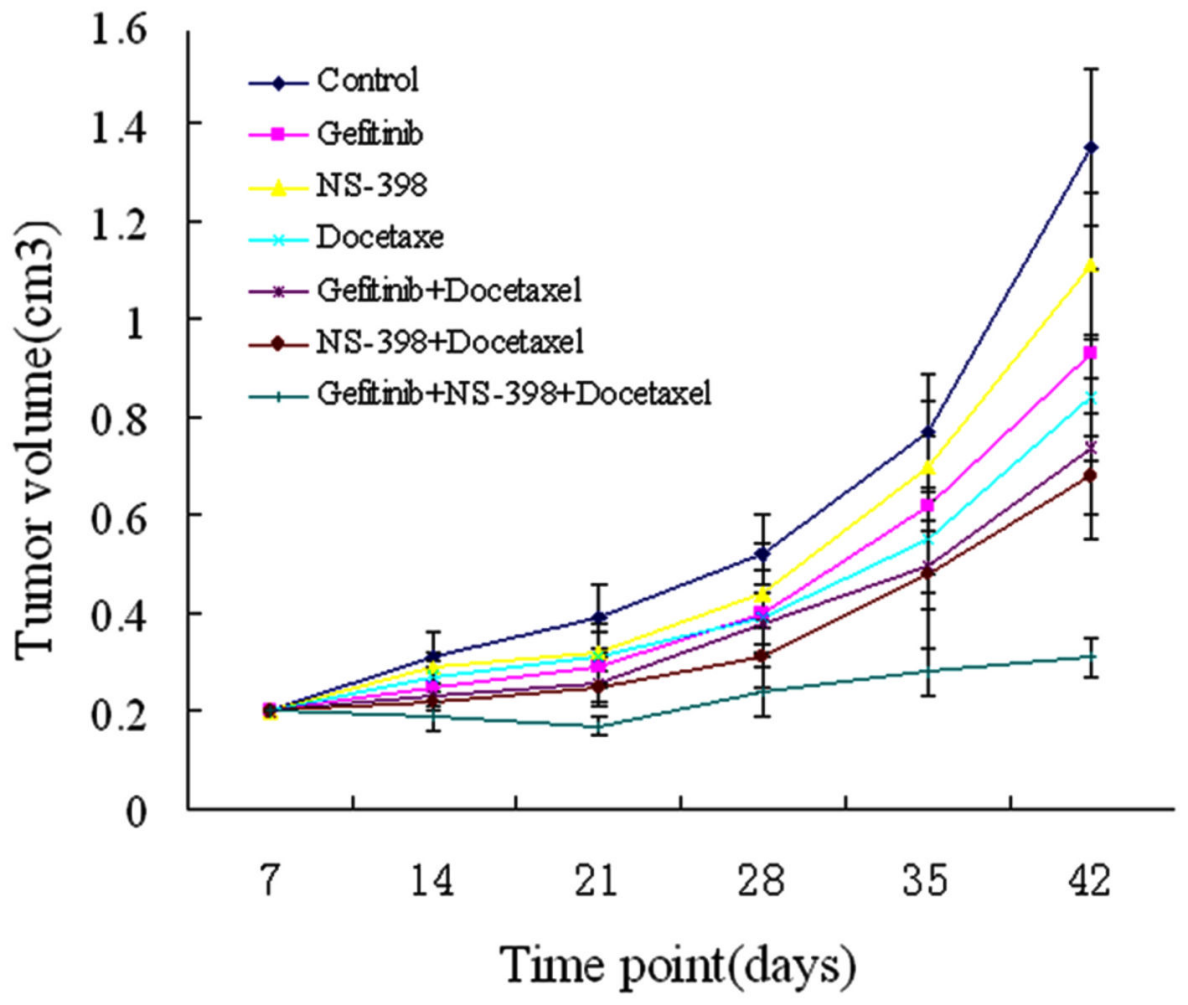
Antitumor activity of docetaxel treatment in combination with gefitinib and NS-398 on established PC-3M human prostate carcinoma xenografts in nude mice. Mice were injected in the dorsal flank with PC3M cells. After 7 days (average tumor size:0.2 cm^3^), mice were treated as described in the Materials and Methods section. Change in tumor volume was evaluated after exposure to Doc, gefitinib and NS-398 alone or in combination. Data are shown as means±SD.

Docetaxel in combination with gefitinib or NS-398 inhibited tumor growth and to a significantly greater extent than in untreated controls or animals treated with a single drug (Figure 7). In these dual combination groups only a modest increase in tumor size was recorded at the end of the experiment 6 weeks after tumor cell injection and 2 weeks after stopping treatment. Combined treatment with any two drugs or with all three drugs was well tolerated; no weight loss or other signs of acute or delayed toxicity were observed.

## Discussion

Docetaxel plays an important role in the treatment of advanced PCa. However, long-term treatment often results in side effects and chemotherapy resistance, resulting in disease recurrence and poor survival. In an attempt to overcome drug resistance, recent studies have focused on low-dosage docetaxel combined with other drugs or on silencing some genes [22-25]. 

EGFR and COX-2 are over-expressed in a number of malignancies including PCa. A growing body of evidence shows that EGFR and COX-2 signaling activities play a crucial role in the development of malignant diseases. Both pathways, therefore, provide attractive targets for anticancer therapy and chemoprevention [5-8,26,27]. Consequently, EGFR and COX-2 inhibitors have been investigated for chemotherapy and cancer prevention [28-31]. 

Over-expression of EGFR and COX-2 and the interaction between EGFR signaling and COX-2 activity have been implicated as causative factors for cancer [32,33]. In our in vitro and in vivo studies, we demonstrated significant up-regulation of EGFR and COX-2 expression in advanced PCa tissue (Table 1) and in corresponding cell lines (Figure 2). It has previously been reported that activation or overexpression of EGFR [12,13] and COX-2 [20] is correlated with docetaxel resistance. This led us to speculate that simultaneously targeting EGFR and COX-2 may be a more effective therapeutic strategy for overcoming docetaxel resistance than targeting either signaling pathway separately.

Our results show that simultaneously blocking EGFR with gefitinib and COX-2 with NS-398 in PCa cells subjected to low-dose docetaxel resulted in a beneficial effect on cell growth inhibition (Figure 3). Median effect analysis using the CI method of Chou and Talalay confirmed a moderately synergistic interaction among these three drugs in both PCa cell lines (Table 2 and 3). In the cell apoptosis and invasion assays the combined effect of the three drugs was slightly greater than additive in the two cell lines. These results are consistent with the findings of the MTT proliferation test, suggesting that this novel combination of drugs may be effective in preventing tumor growth and distant metastases. 

We also demonstrated that the three drug combination was associated with marked cell apoptosis (Figure 4) and loss of invasive ability (Figure 5). In a previous study gefitinib had no single-agent activity, but its combination with docetaxel was associated with the same response rate in advanced PCa as docetaxel monotherapy [7]. It has also been shown that cyclooxygenase-2 inhibition augments docetaxel-induced apoptosis [17] which is consistent with the results in our study. However, long–term, high-dose therapy with this combination is associated with toxicity and side-effects that prevents its use in clinical practice. High dose docetaxel is associated with nervous system injury, hematopoietic repression and chemoresistance which makes it un suitable for many patients with advanced disease 

Importantly, we showed that adding an EGFR inhibitor and COX-2 specific inhibitor to low-dose docetaxel regimens may provide a strategy for reducing dose-related side-effects. Simultaneously targeting EGFR and COX-2 appeared to sensitize PCa cells to the effects of docetaxel in vitro. Our results in vivo were consistent with these in vitro findings, indicating that combined treatment is superior to single- or double–agent therapy. The combination of any two drugs, or all three drugs, was well tolerated. No acute or delayed toxicity was observed in any of the animals. Thus, the combination of gefitinib, NS-398 and docetaxel may represent a promising new approach for the treatment of advanced PCa.

NF-ΚB transcription factor is constitutively activated in PCa cells and is thought to contribute to the development and progression of PCa. Increasing evidence suggests that NF-ΚB activation and upregulation has the potential to reduce the anti-tumor effects of docetaxel. Inhibition of NF-ΚB therefore appears to be a promising new therapeutic strategy [34,35]. NF-ΚB is normally retained in an inactive form in the cell cytoplasm, where it is bound by inhibitory proteins. After activation, NF-ΚB is released from its inhibitor and translocated from the cytoplasm to the nucleus, where it binds to cognate sequences in the promoter region of many target genes. 

In the present study, the mRNA expression of p65 (a subunit of NF-ΚB which translocates to the nucleus) was down-regulated to a greater extent after incubation with the three-agent combination treatment than after exposure of either cell line to docetaxel alone (Figure 6A and B). In addition, p65 protein expression was down-regulated in the PC-3M cell line (Figure 6C). however, further investigations are required to clarify the mechanisms involved in this process.

The process of angiogenesis is integral to cancer progression, invasion and metastasis. VEGF is a highly effective pro-angiogenesis factor that is believed to participate in the development of PCa and in its metastasis [36,37]. Inhibition of VEGF has been shown to enhance the antitumor effect of docetaxel in patients with advanced PCa [38,39]. Cancer invasion and metastasis involves cellular detachment, transport through the extracellular matrix and degradation of the basement membrane, in a series of processes that depend on matrix metalloproteinase (MMP). Previous studies have shown that MMP-9 is up-regulated during docetaxel treatment and that inhibition of MMP-9 enhances it anti-tumor effect [40,41]. 

In the present study we demonstrated a significant decrease in cell migration in vitro after exposure to the three-agent combination. We also showed that the same three-agent combination inhibited VEGF and MMP-9 activity at both mRNA and protein levels in PC-3M cells (Figure 6A and C). However, no significant variation in levels of MMP and VEGF was observed in DU-145 cells (Figure 6B and D). Based on these findings it is possible that gefitinib, NS-398 and docetaxel work together to inhibit the expression and secretion of VEGF and MMP-9, thus hindering invasion and reducing the metastasis potential of PC-3M cells. Further research is underway to elucidate the exact mechanism involved in this process

## Conclusions

Taken together our results suggest that EGFR and COX-2 play an important role in the sustained growth, survival, and invasion of PCa cells. The combined usage of docetaxel with selective inhibitors of EGFR and COX-2, such as gefitinib and NS-398, represents a promising strategy for the treatment of advanced PCa. This novel combination of drugs offers a new choice for current docetaxel-based regimens and may constitute a rational basis for future clinical trials to evaluate its efficacy and safety.
